# Comparing antibiotic prescribing patterns for hidradenitis suppurativa between the emergency department and ambulatory care setting

**DOI:** 10.1371/journal.pone.0310651

**Published:** 2025-05-19

**Authors:** Hannah Tolson, Rebecca K. Yamamoto, Robin Kikuchi, Kaviyon Sadrolashrafi, Audrey Hao, Lily Guo, Sara Bilimoria, Danielle Yee, April W. Armstrong

**Affiliations:** 1 University of Arizona College of Medicine, Phoenix, Arizona, United States,; 2 Georgetown University School of Medicine, Washington, District of Columbia, United States,; 3 Keck School of Medicine of University of Southern California, Los Angeles, California, United States,; 4 Kirk Kerkorian School of Medicine at UNLV, Las Vegas, Nevada, United States,; 5 Duke University School of Medicine, Durham, North Carolina, United States,; 6 Division of Dermatology, Department of Medicine, David Geffen School of Medicine at the University of California, Los Angeles, California, United States; Università degli Studi di Milano, ITALY

## Abstract

**Background:**

Oral antibiotics are a mainstay of treatment for hidradenitis suppurativa (HS), primarily due to their anti-inflammatory and anti-microbial properties. There is a paucity of literature comparing how antibiotic prescribing patterns for HS patients differ between the emergency department (ED) and ambulatory care settings.

**Objective:**

This study aims to compare antibiotic prescribing patterns for HS patients in the ED versus ambulatory care setting.

**Methods:**

We utilized the National Ambulatory Medical Care Survey (NAMCS) and the National Hospital Ambulatory Medical Care Survey (NHAMCS) to identify visits for HS patients in 2005-2016, 2018, and 2019. We performed multivariate logistic regression analysis to compare the likelihood of prescribing antibiotic therapy versus no antibiotic therapy between the ED and ambulatory care settings.

**Results:**

We identified a weighted total of 3,041,193 HS patient visits. Approximately 49.0% of ambulatory visits resulted in antibiotic prescriptions. The most frequently prescribed antibiotics in the ambulatory setting were tetracyclines (41.2%), clindamycin (35.9%), and trimethoprim/sulfamethoxazole (21.6%). Approximately 74.7% of ED visits resulted in antibiotic prescriptions. The most frequently prescribed antibiotics in the ED setting were trimethoprim/sulfamethoxazole (44.5%), beta-lactams (33.2%), and clindamycin (27.7%). Multivariate logistic regression demonstrated significantly higher odds of receiving antibiotics in ED visits compared to ambulatory care visits. (OR 3.88; 95% Cl, 1.28-11.77; *p* = 0.02).

**Conclusion:**

Antibiotic class selection varied significantly between the ED and ambulatory settings. Additionally, ED visits were more likely to result in antibiotic prescriptions than ambulatory visits for HS patients.

## Introduction

Hidradenitis suppurativa (HS) is a chronic, debilitating skin disease that affects approximately 0.1% of the US population [1]. Hidradenitis suppurativa is characterized by inflammatory skin lesions in apocrine gland-bearing regions [2]. Patients with HS experience recurrent, painful flares that resemble soft tissue infections [3]. Due to severe pain and concern for infection, HS patients often seek care in the emergency department (ED). Consequently, HS is one of the most frequently treated chronic dermatological diseases encountered in the ED [3,4].

Oral antibiotics are one of the main treatments for HS, primarily due to their anti-inflammatory and anti-microbial properties [5]. The exact pathophysiology of HS is poorly understood, and the role of bacteria in HS is widely debated. While HS is primarily an immune-mediated disease, microflora colonizing HS lesions may exacerbate the inflammatory cycle [1]. Thus oral tetracyclines, which can suppress inflammation, are a first-line oral antibiotic treatment for mild-to-moderate HS (Level II, III recommendation made by the North American Clinical Management Guidelines for Hidradenitis Suppurativa) [2,6]. Other oral antibiotics are used in combination therapy regimens, such as rifampin plus clindamycin [7,8]. However, there is limited evidence to support the use of oral antibiotic monotherapy, besides tetracyclines, to treat HS [2].

Patients with HS are often prescribed longer antibiotic courses and show increased resistance to standard antibiotics, even though their abscesses are often sterile [9,10]. Therefore, antibiotic stewardship is an important consideration in HS. Despite the high frequency of HS treatment in the ED and ambulatory care settings, there is a paucity of literature comparing how antibiotic prescribing patterns differ between them. This study aims to compare antibiotic prescribing patterns for HS patients in the ED versus ambulatory visit setting.

## Methods

We utilized data from the National Ambulatory Medical Care Survey (NAMCS) and the National Hospital Ambulatory Medical Care Survey (NHAMCS) for this cross-sectional study. The data was accessed on October 13^th^, 2024, and information that could identify individual participants during or after data collection was not available. We identified visits for patients with HS from 2005-2016, 2018, and 2019. The NAMCS and the NHAMCS provide estimates of a nationally representative sample of patient visits using a complex probability survey design with masked weighting variables; using these weighted samples is recommended to ensure accurate analysis [11]. Physicians participating in the NAMCS and the NHAMCS were randomly selected and asked to collect information on their practice characteristics [12]. This study used publicly available data and therefore did not require Institutional Review Board approval.

We included visits for patients of all ages in the United States with a diagnosis of HS between January 1, 2006, and December 31, 2019. Data from the year 2017 was unavailable and therefore, not included. We identified visits for HS patients using the *International Classification of Diseases, Ninth Revision* (*ICD-9*) code 70583, or the *International Classification of Diseases, Tenth Revision* (*ICD-10*) code L732.

The outcome variable was the presence of antibiotic prescriptions. We defined antibiotic prescriptions as prescriptions for any of the following medications: tetracyclines, cephalosporins, penicillins, clindamycin, fluoroquinolones, trimethoprim/sulfamethoxazole (TMP/SMX), rifampin, dapsone, macrolides, or vancomycin. Patients who were prescribed multiple antibiotics were counted once in the antibiotic group. Patients seen in the emergency department were included in the antibiotic group if they were prescribed the antibiotic at discharge. For frequency analysis, we combined cephalosporins and penicillins into a “beta-lactams” group due to small sample size.

The independent variable was the healthcare visit setting, which was characterized as (1) the ED setting and (2) the ambulatory care setting.

We calculated descriptive statistics for patient demographics, clinical characteristics, and patient outcomes. Continuous variables were reported with mean and standard deviation. Categorical variables were reported with (weighted) raw numbers and proportions. We performed frequency counts for antibiotic prescriptions for (1) the total population and (2) the total population stratified by visit setting (ED vs. ambulatory care setting). We performed multivariate logistic regression using the presence of antibiotic prescriptions as the outcome variable and the visit setting (ED vs. ambulatory care setting) as the independent variable. The logistic regression model was adjusted for age, sex, insurance type, race, and medical comorbidities (measured by the Charlson Comorbidity Index). We set the threshold for significance at a p-value less than 0.05. We performed all data management and analyses with Stata 18.0 statistical software.

## Results

We identified a weighted total of 3,041,193 HS patient visits. Approximately 87.3% of all patient visits were in the ambulatory care setting and 12.7% were in the ED setting. The sociodemographic characteristics of these visits are available in Table 1.

**Table 1:** Sociodemographic characteristics for overall visits, ambulatory visits, and emergency department visits.

Characteristic	Overall Visits weighted n = 3,041,193	Ambulatory Visits weighted n, (%) = 2,655,635 (87.3)	ED Visits weighted n, (%) = 385,558 (12.7)	*P*-value
**Antibiotics, n (%)** **-prescribed** **-not prescribed**	1,588,399 (52.2) 1,452,794 (47.8)	1,300,543 (49.0) 1,355,092 (51.0)	287,856 (74.7) 97,702 (25.3)	<0.0001
**Sex, n (%)** **-Male sex** **-Female sex**	833,753 (27.4) 2,207,440 (72.6)	729,519 (27.5) 1,926,116(72.5)	104,234 (27.0) 281,324 (73.0)	0.083^†^
**Age, mean (SEM) years**	37.0 (1.5)	38.0 (1.6)	29.8 (2.0)	0.0006^*^
**Insurance, n (%)** **-Private** **-Medicare** **-Self-pay** **-Medicaid or CHIP** **-Other/ Unknown**	1,624,365 (53.4) 281,124 (9.3) 73,969 (2.4) 873,815 (28.7) 187,920 (6.2)	1,514,597 (57.0) 242,984 (9.1) 38,268 (1.5) 717,329 (27.0) 142,457 (5.4)	109,769 (28.5) 38,140 (9.9) 35,700 (9.2) 156,486 (40.6) 45,463 (11.8)	<0.0001^†^
**Race/Ethnicity, n (%)** **-White Only** **-Black Only** **-Hispanic** **Other Race/Multiple Race**	1,897,298 (62.4) 833,641 (27.4) 267,095 (8.8) 43,159 (1.4)	1,753,450 (66.0) 670,171 (25.2) 199,255 (7.5) 32,759 (1.2)	142,848 (37.3) 163,469 (42.4) 67,840 (17.6) 10,401 (2.7)	0.014^†^
**CCI, mean (SEM)**	0.4 (0.1)	0.4(0.1)	0.03 (0.02)	0.0015^*^
**MSA status, n (%)** **-Urban** **-Rural**	2,851,363 (93.8) 189,830(6.2)	2,488,410 (93.7) 167,225 (6.3)	362,953 (94.1) 22,605 (5.9)	0.48^†^

* Analysis of variance of the differences among visits with HS patients of different ages and Charlson Comorbidity Indices (CCI).

† X^2^ test of the differences among visits with HS patients of different races, insurance types, and sexes.

**Fig 1:**
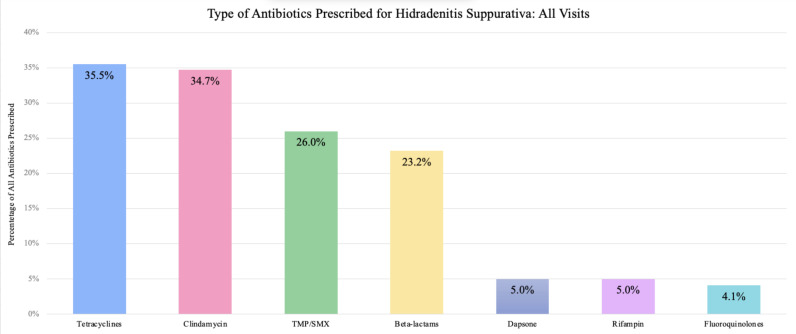
Bar graphs demonstrating the frequency of each antibiotic prescription for all HS visits.

Overall, 52.2% of HS visits involved patients who were prescribed antibiotics. Tetracyclines were prescribed most frequently (35.5%), followed by clindamycin (34.7%), TMP/SMX (26%), beta-lactams (23.2%), rifampin (5.0%), dapsone (5.0%), then fluoroquinolones (4.1%) (Fig 1).

Approximately 49.0% of ambulatory care visits resulted in antibiotic prescriptions. The most frequently prescribed antibiotics in the ambulatory care setting were tetracyclines (41.2%), clindamycin (35.9%), TMP/SMX (21.6%), beta-lactams (20.7%), rifampin (5.9%), dapsone (5.9%), and fluoroquinolones (4.4%).

**Fig 2:**
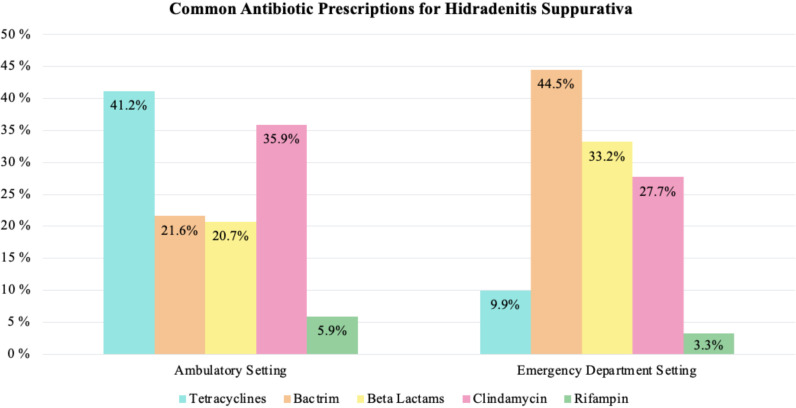
Bar graphs demonstrating the most frequently prescribed antibiotics by visit setting.

Approximately 74.7% of ED visits resulted in antibiotic prescriptions. The most frequently prescribed antibiotics in the ED setting were TMP/SMX (44.5%), beta-lactams (33.2%), clindamycin (27.7%), tetracyclines (9.9%), rifampin (3.3%), dapsone (3.3%), and fluoroquinolones (3.0%). The top 5 most frequently prescribed antibiotics by visit setting are shown in Fig 2.

**Fig 3:**
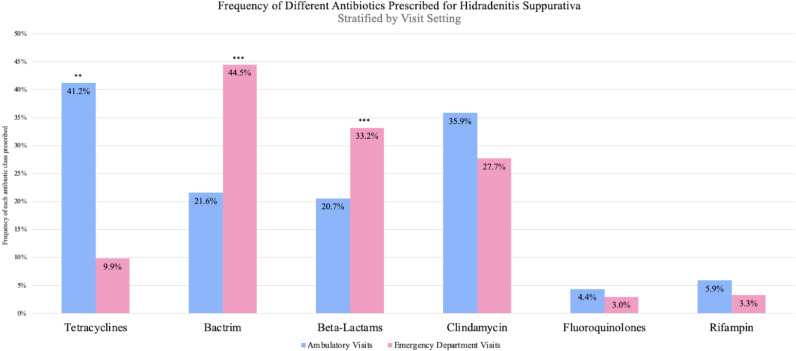
Bar charts demonstrating the frequency of different antibiotic prescriptions, stratified by visit setting. P-values less than 0.01 on chi-squared testing are summarized with two asterisks, while p-values less than 0.001 on chi-squared testing are summarized with three asterisks.

We compared prescription rates between commonly prescribed antibiotics in the ED and ambulatory care settings. Chi-squared testing demonstrated a statistically significant difference in the frequency of antibiotic prescriptions between the ED and ambulatory care settings for tetracyclines (P = 0.004), TMP/SMX (P < 0.0001), and beta-lactams (P < 0.0001). Comparisons of the antibiotic class prescribed by visit setting are demonstrated in Fig 3.

**Fig 4:**
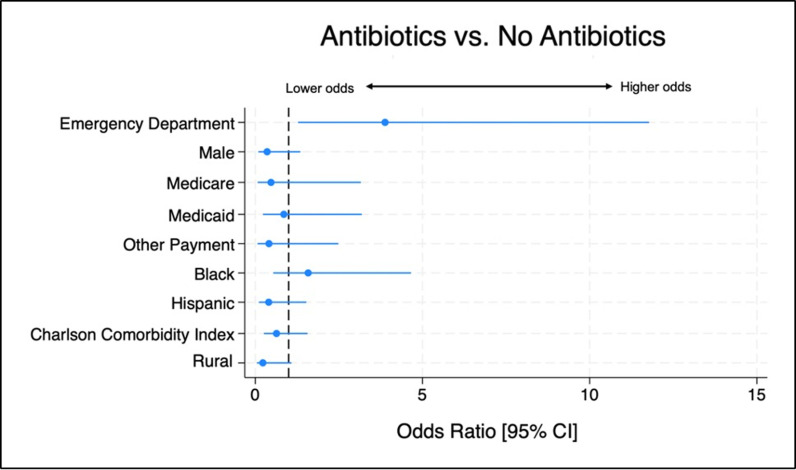
Odds ratios of the probability of antibiotic prescriptions in HS patient visits, adjusted for visit setting (ref: ambulatory), sex (ref: female), age, insurance type (ref: private), race/ethnicity (ref: white), CCI, and rural status (ref: urban).

We performed multivariate logistic regression analysis to compare the difference in the likelihood of receiving antibiotics between the ED and the ambulatory care setting. There was a significantly greater likelihood of receiving antibiotics in the ED compared to the ambulatory visit setting (OR 3.88; 95% Cl, 1.28-11.77; *p* = 0.02) (Table 2). The forest plot of the multivariable logistic regression analysis results is presented in Fig 4.

**Table 2:** Multivariate logistic regression analysis of the association between antibiotic prescriptions and visit setting in HS patient visits, adjusting for sex, race, insurance type, age, rural/urban status, and Charlson Comorbidity Index.

Antibiotics vs. No Antibiotics	OR (95% CI)	P value
**Visit type** Ambulatory Emergency Department	(Ref) 3.88 (1.28- 11.77)	– 0.02
**Sex** Female Male	(Ref) .35 (0.09- 1.35)	– 0.13
**Age**	1.02 (0.98 – 1.07)	0.25
**Insurance type** Private Insurance Medicare Self-pay Medicaid Other	(Ref) 0.47 (0.07-3.15) 1.92 (0.28-13.03) 0.86 (0.23- 3.18) 0.41 (0.07- 2.48)	– 0.43 0.50 0.81 0.33
**Race** White Black Hispanic Other Race	(Ref) 1.58 (0.54-4.66) 0.40 (0.11- 1.53) 1.37 (0.16- 11.6)	– 0.40 0.18 0.77
**Charlson comorbidity index**	0.64 (0.26 – 1.56)	0.32
**Rural/urban status** Urban Rural	(Ref) .23 (0.05- 1.08)	– 0.06
**F(12, 46)**	1.49	
**Prob > F**	0.17	

## Discussion

This retrospective, cross-sectional study is a pioneering study to examine antibiotic prescribing patterns in different medical settings for the treatment of HS. We found a significantly higher likelihood of receiving antibiotic prescriptions in the ED compared to the ambulatory care setting (Table 2). Additionally, the antibiotic classes selected in the ambulatory care setting varied significantly from those selected in the ED setting.

One possible explanation for these findings is that emergency physicians are more likely to manage HS as soft tissue infections. Soft tissue infections (e.g., furuncles, carbuncles, and abscesses) are commonly encountered in the ED and universally treated with antibiotics [13]. Patients experiencing HS flares present with symptoms similar to soft tissue infections, such as painful, erythematous abscesses with suppurative discharge [14]. Therefore, even when ED physicians accurately diagnose HS, some may treat HS as a soft-tissue infection, which may explain the high rates of antibiotic prescription among ED physicians.

We also note that different antibiotics were prescribed between the ED and the ambulatory setting. This further supports the idea that HS is more likely to be managed as soft tissue infections in the ED. The three most prescribed antibiotics in ED visits were TMP/SMX, beta-lactams, and clindamycin, which are commonly selected for soft tissue infection [15]. In contrast, tetracyclines were seldom prescribed despite being widely considered the first-line antibiotic choice for HS. These results further support the explanation that HS patients seen in the ED are likely to receive treatment for a diagnosis of soft tissue infections rather than HS. Prior dermatology literature supports this hypothesis: a survey of emergency room doctors found that HS patients were treated for bacterial infection/furunculosis in the ED 40.2% of the time [16].

We observed a substantial variation in the selection of antibiotics across the entire population, suggesting that unfamiliarity with treating HS may extend to the ambulatory setting. Many specialties were represented in the ambulatory cohort, including dermatology, internal medicine, family medicine, ob-gyn, general surgery, and urology. However, each of these specialties has varying levels of experience in treating skin diseases. For instance, in a 2021 survey of 211 family medicine physicians, only 23.7% reported feeling confident in diagnosing HS, and 63% defined HS as an infectious process of apocrine glands [17]. Taken together, our results suggest a global lack of knowledge and/or limited adherence to HS treatment guidelines in both the ED and ambulatory settings.

The findings of this study should be interpreted in the context of the study design. The NAMCS database does not include longitudinal treatment data, so we could not evaluate the length of time for which each antibiotic was prescribed. Additionally, the severity of the disease cannot be ascertained in most large database studies. Although it would be preferable to utilize the severity of HS in our analysis, we could not stratify our analysis by disease severity, as NAMCS does not capture severity using validated measures.

Consistent management in the ambulatory setting decreases the frequency of HS flares and the need to utilize ED services [18]. Since oral antibiotic therapy remains a popular treatment for HS, clinicians must carefully consider their choice of antibiotic class and dosing frequency to avoid resistance. To this end, efforts have been made to disseminate standardized information about treating HS, including the introduction of national guidelines [8]. Additionally, newer therapeutic options, like biologics (adalimumab and secukinumab), show great promise for the management of moderate to severe HS [19]. Utilizing these biologics has the potential to limit HS flares and ED visits, thereby globally reducing antibiotic prescriptions [20]. Future work may be directed toward understanding antibiotic prescribing patterns among different ambulatory specialties to further assess the differences in prescribing patterns for HS among dermatologists versus non-dermatologists.

## Conclusion

In conclusion, ED visits were more likely to result in antibiotic prescriptions than ambulatory care visits for HS patients. Antibiotic class selection in the ED suggests that a part of those cases of HS are treated as soft-tissue infections. These results indicated that visit setting is a significant determinant of antibiotic therapy selection for HS patients in the United States. The goal of HS treatment should be directed toward improving disease control in the ambulatory setting so that patients need to access the ED less frequently.
